# Inspiratory pressure waveform influences time to failure, respiratory muscle fatigue, and metabolism during resistive breathing

**DOI:** 10.14814/phy2.15668

**Published:** 2023-05-05

**Authors:** Mathias Krogh Poulsen, Stephen E. Rees, John Hansen, Andrew J. T. Stevenson, Søren Kjærgaard, Carlo A. Volta, Savino Spadaro, Dan S. Karbing

**Affiliations:** ^1^ Respiratory and Critical Care Group, Department of Health Science and Technology Aalborg University Aalborg Denmark; ^2^ CardioTech, Department of Health Science and Technology Aalborg University Aalborg Denmark; ^3^ Neural Engineering and Neurophysiology, Department of Health Science and Technology Aalborg University Aalborg Denmark; ^4^ Department of Anesthesiology University Hospital Aalborg Denmark; ^5^ Intensive Care Unit, Morphology Surgery and Experimental Medicine University of Ferrara Ferrara Italy

**Keywords:** muscle fatigue, resistive breathing, respiration pattern, respiratory muscles

## Abstract

Increased ventilatory work beyond working capacity of the respiratory muscles can induce fatigue, resulting in limited respiratory muscle endurance (*T*_lim_). Previous resistive breathing investigations all applied square wave inspiratory pressure as fatigue‐inducing pattern. Spontaneous breathing pressure pattern more closely approximate a triangle waveform. This study aimed at comparing *T*_lim_, maximal inspiratory pressure (PI_max_), and metabolism between square and triangle wave breathing. Eight healthy subjects (*Wei* = 76 ± 10 kg, *H* = 181 ± 7.9 cm, age = 33.5 ± 4.8 years, sex [F/M] = 1/7) completed the study, comprising two randomized matched load resistive breathing trials with square and triangle wave inspiratory pressure waveform. *T*_lim_ decreased with a mean difference of 8 ± 7.2 min (*p* = 0.01) between square and triangle wave breathing. PI_max_ was reduced following square wave (*p* = 0.04) but not for triangle wave breathing (*p* = 0.88). Higher VO_2_ was observed in the beginning and end for the triangle wave breathing compared with the square wave breathing (*p* = 0.036 and *p* = 0.048). Despite higher metabolism, *T*_lim_ was significantly longer in triangle wave breathing compared with square wave breathing, showing that the pressure waveform has an impact on the function and endurance of the respiratory muscles.

## Graphical abstract

Previous resistive breathing investigations applied square wave inspiratory pressure as fatigue‐inducing breathing pattern, even though spontaneous breathing pressure pattern more closely approximates a triangle waveform. During randomized square or triangle wave breathing trials, respiratory endurance increased during triangle wave breathing by 22% (*p* = 0.01). In conclusion, the inspiratory pressure waveform has a significant impact on the function and endurance of the respiratory muscles.

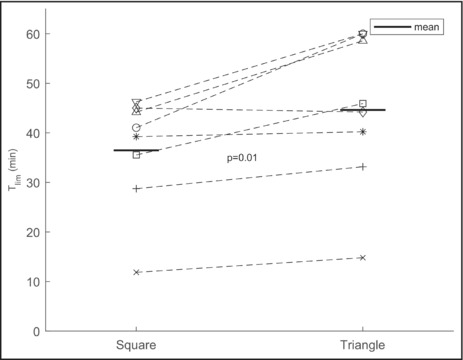

## 1 INTRODUCTION

Ventilatory work increase beyond working capacity of the respiratory muscles can induce fatigue, reducing the working capacity of the respiratory system which in turn can limit gas exchange and exercise performance. Respiratory muscle fatigue (RMF) has been shown to be reversible by rest (Workshop, 1990), but the occurrence of RMF has immediate consequences during exercise, such as decreased peripheral blood flow to exercising musculature (Dempsey et al., 2006), and in patients with lung disease and during mechanical ventilation where RMF can occur during weaning (Laghi et al., 2003) consequently leading to hypercapnic respiratory failure (Cohen et al., 1982).

RMF can occur after intense exercise in healthy individuals due to the high respiratory demands occurring during full‐body exercise (Janssens et al., 2013; Johnson et al., 1993; Verges et al., 2006). In patients suffering from prolonged respiratory failure, RMF can occur because of either pathological conditions or weakened respiratory muscle function (Cohen et al., 1982; Vassilakopoulos et al., 1998). Specifically, studies have shown that maximal pressure generation of the respiratory muscles decreases following exercise or loaded respiratory work (Dempsey et al., 2006), clearly demonstrating that RMF can occur in respiratory demanding situations.

Use of esophageal and gastric pressure catheters for monitoring of respiratory muscle work is considered the reference technique when evaluating RMF. The techniques for measuring RMF can involve maximal contraction attempts by peripheral electrical stimulation of the phrenic nerves or cervical magnetic stimulation (CMS) of the respiratory muscles. Measurement of esophageal pressure (*P*_es_) is applied for assessing the work of the combined respiratory muscles, where the work of the diaphragm can be elucidated by both measuring *P*_es_ and gastric pressure (*P*_ga_) to deduct the transdiaphragmatic pressure (*P*_di_ = *P*_ga_ − *P*_es_) (American Thoracic Society/European Respiratory Society, 2002; Similowski et al., 1989). RMF is then assessed from the relationship between maximal and imposed load (American Thoracic Society/European Respiratory Society, 2002). Roussos and Macklem showed that breathing at a load >40% of maximal inspiratory diaphragmatic pressure (*P*_di_/*P*_di,max_) led to RMF within 45 min showing a finite endurance at these loads. They also demonstrated a reduction in respiratory muscle endurance with increasing breathing load (Roussos & Macklem, 1977). Conversely, Bellemare and Grassino (1982) showed that below a certain threshold, subjects could maintain a given load indefinitely, or at least 45 min as defined by these investigators.

Increasing duty cycle of the respiratory muscles, defined as an increased ratio of inspiratory to total breath time (*T*_i_/*T*_tot_), also reduces respiratory muscle endurance. Bellemare and Grassino (1982) showed that the tension time index (TTI), which is the product of *T*_i_/*T*_tot_ and *P*_di_/*P*_di,max,_ could describe muscle endurance. They showed that if TTI < 0.15, breathing can be maintained indefinitely (>45 min). Above this threshold, respiratory muscle endurance will be progressively reduced.

Respiratory muscle endurance has also shown to be compromised by increasing breathing frequency (Dodd et al., 1988), hyperventilation (Tzelepis et al., 1988), and respiratory muscle metabolism (Collett et al., 1985; Field et al., 1984; McCool et al., 1989). However, it is typical for RMF to be assessed from duty cycle and diaphragmatic inspiratory pressure components as expressed in the TTI, and as proposed by Bellemare and Grassino.

Common to all previous studies of respiratory muscle endurance is the application of resistive breathing as a loading modality applying different variations of tidal volume (*V*_t_), duty cycle, and level of load. To the best of the authors' knowledge, all previous studies have applied a square inspiratory pressure waveform as the load breathing pattern for inducing fatigue. This is problematic, as inspiratory pressure waveforms during inspiration are not typically square wave, but more closely approximate a triangle shape (Benditt, 2005), potentially because the respiratory musculature operates most efficiently at this given modality. The question therefore arises whether the current understanding of the effect of load, duty cycle, etc. truly reflects the endurance of the respiratory muscles during natural breathing pressure patterns.

This study investigates RMF in healthy subjects breathing with both square and triangle inspiratory pressure patterns. The purpose of the study is to compare respiratory muscle endurance, metabolic parameters, and perceived effort during loaded breathing with either a square wave or a triangle wave pressure waveform and further, to investigate whether RMF is evident as a consequence of the loaded breathing.

## 2 MATERIALS AND METHODS

### 2.1 Participants

Twelve healthy subjects (*W* = 74 ± 9 kg, *H* = 179 ± 7 cm, age = 33.5 ± 9 years, sex [F/M] = 2/10) were enrolled after written informed consent. Subjects were considered healthy if they had body mass index between 20 and 30 kg/m^2^ and were non‐smokers and had no history of neuromuscular, circulatory, or respiratory disease. Furthermore, pregnancy led to exclusion due to safety reasons. Subjects were asked to refrain from strenuous exercise <48 h before any experimentation and further to avoid eating 2 h prior to the experiment.

### 2.2 Experimental design

**FIGURE 1:**
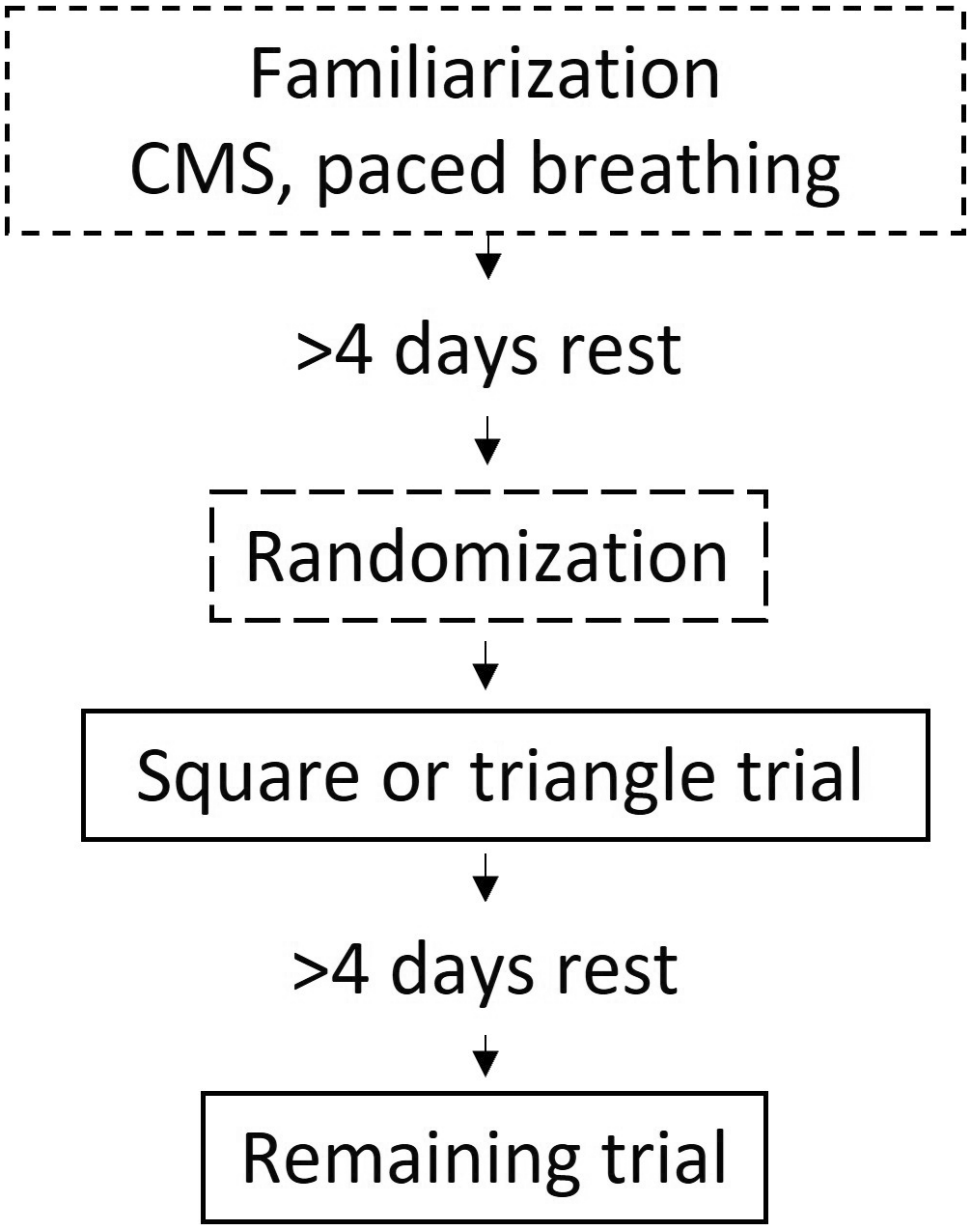
Overview of the study. Dashed line boxes signify familiarization and randomization. The solid black boxes show the two trials.

This study was a cross‐over randomized and single‐blinded trial where subjects attended the laboratory on three occasions, all separated by >4 days of rest, as can be seen in Figure 1.

First, subjects underwent a familiarization trial where different breathing patterns were followed for a minimum of 20 min including maximal inspiratory pressure effort attempts.

After this, subjects were randomized to square and triangle wave breathing trials. During each trial, subjects were seated upright in a chair with armrests. Maximal inspiratory esophageal pressure (PI_max_) was measured by a minimum of three maximal inspiratory pressure attempts without and three with CMS superimposed on top of a volitional PI_max_, all separated by 1 min. Maximal inspiratory pressure attempts were performed from functional residual capacity as indicated by *P*_es_ = 0 cmH_2_O after relaxed exhalation. The subjects were instructed to inspire as hard as possible for 3 s only with the diaphragm against an occluded airway (Mueller maneuver) and not raise the shoulders and ribcage. PI_max_ was noted as the highest peak *P*_es_ value achieved at any of the maximal inspiratory pressure attempts.

**FIGURE 2:**
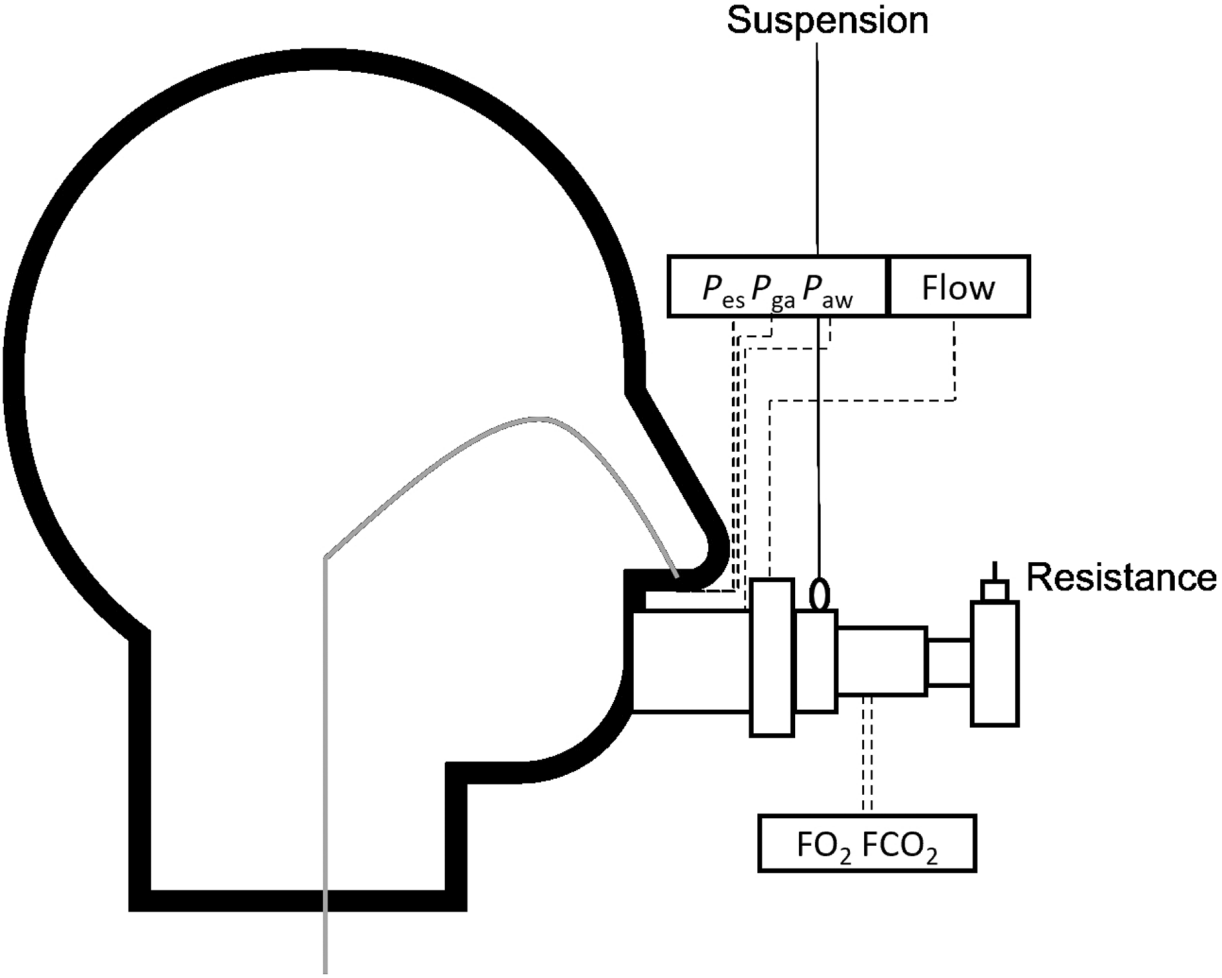
Figure shows a sketch of the measurement tubes, suspension, orifice resistance, and esophageal catheter. The dotted lines indicate measurement tubes connecting to the measurement devices.

Target pressures for the square and triangle breathing patterns were set as to target a TTI of 0.25 for both patterns. This was achieved by adding an inspiratory orifice resistance of 2–2.5 mm (TB90, Dolema) to the inspiratory side of a standard two‐way valve (T‐piece directional valve, Intersurgical) (see Figure 2). The square wave mean *P*_es_ was set to 50% of individual PI_max_. The target pressure for triangle wave breathing was a peak of 90% PI_max_, corresponding to a mean of 50% PI_max_ during inspiration.

**FIGURE 3:**
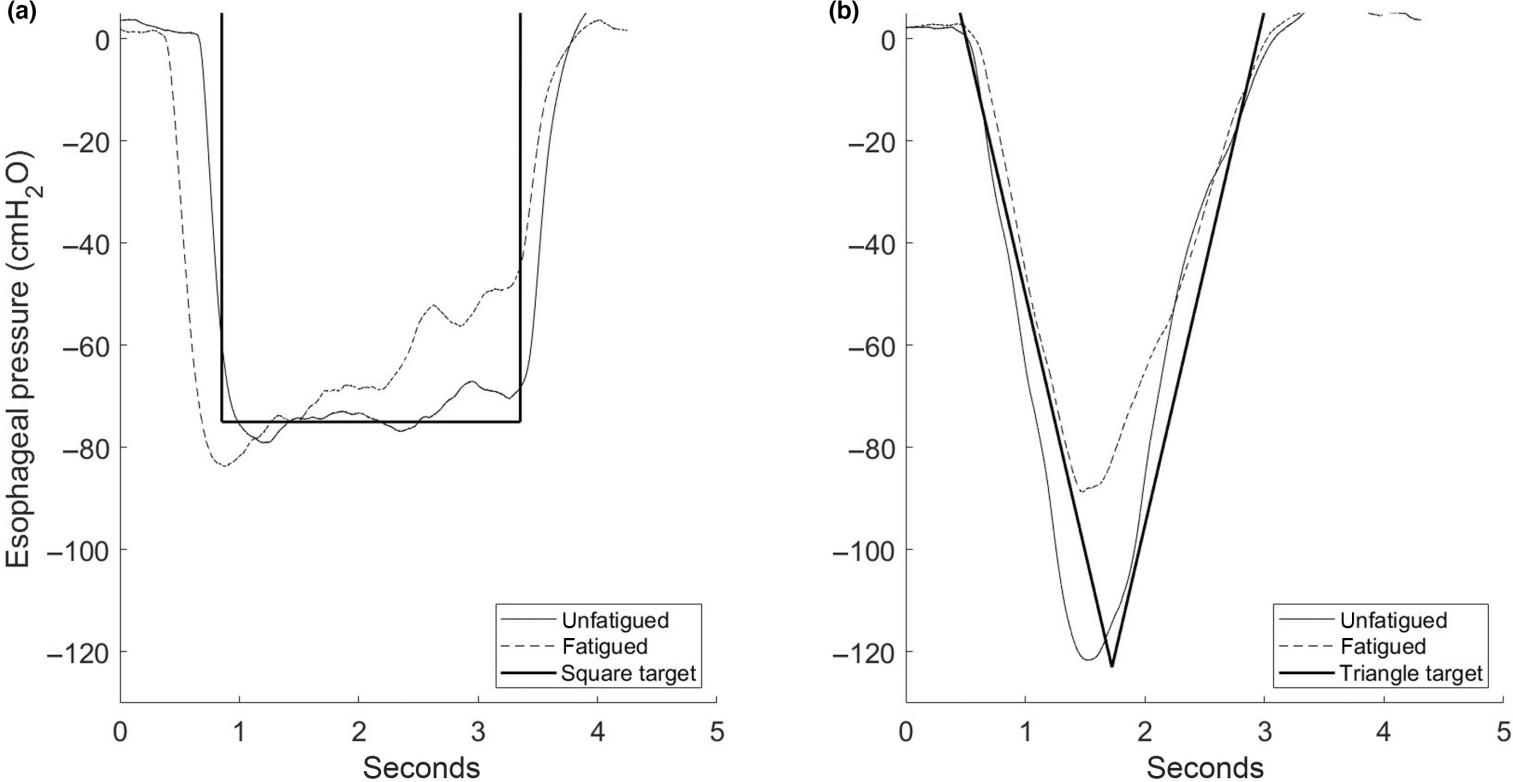
Square wave (a) and triangle wave (b) esophageal pressure trace from one unfatigued breath (solid line) in the beginning of a trial and one fatigued breath (dashed line) in the end. Included is the target trace line which the subject had to follow (bold black line).

The subjects had to follow either a square or a triangle pressure waveform showed on a computer screen in real‐time with custom‐written software (LabView 2020, National Instruments). For both trials, the breathing frequency was set to 12 breaths min^−1^ with an inspiratory to expiratory ratio of 1:1. The guide trace for the two breathing patterns can be seen in Figure 3.

To secure uniformity within and between trials, the elbows of the subjects were positioned on the armrest of the chair while the subjects placed their hands on a suspended mouthpiece. The experimenter instructed the subject not to contract inspiratory accessory muscles or leg muscles and confirmed this during experiments by visual inspection and occasional palpation. The subject was allowed to briefly detach from the mouthpiece to clear the mouth of any excess saliva when necessary.

The subject was asked to continue breathing until the experimenter ended the trial or until volitional exhaustion. The experimenter evaluated inability to continue the breathing task from visual inspection of the online *P*_es_ measurement and guide trace. Inability to continue the breathing task was defined as at least three consecutive breaths where the target pressure could not be maintained despite vigorous verbal encouragement. This evaluation was blinded to the subject. If the subject was breathing past 60 min, the trial was ended as this was considered as ability to breathe indefinitely.

After trial termination, PI_max_ was again measured as outlined above.

### 2.3 Pressure measurements

For square and triangle wave breathing, subjects were fitted with a double‐balloon nasogastric catheter (NitriVent, SIDAM). The catheter was prepared according to manufacturer specifications and the subject was prepared with a local sedative in the nasal cavity (Xylocain Gel 2%, Aspen Pharmacare). The catheter was initially inserted 42.5 cm and both balloons were inflated with 4 mL of air. Catheter position was validated by assuring presence of negative swings for *P*_es_ and positive swings for *P*_ga_ during a forced inspiration (Akoumianaki et al., 2014). If the *P*_es_ trace was positive during inspiration, the catheter was withdrawn in steps of 1 cm until negative swings appeared. If the *P*_es_ trace was influenced by cardiogenic oscillation, the catheter was inserted in steps of 1 cm, until absence of oscillation. Once in place, the ratio of Δairway pressure/Δesophageal pressure during inspiration against an occluded airway was confirmed close to unity with a max of ±20% and if not, the balloons were filled with additional1 mL of air until the threshold was reached (Akoumianaki et al., 2014). A wax nose‐stop plug (Wax earplugs, Quies) was attached to the catheter to avoid air leaks and the subject was fitted with a nose clip for catheter fixation (Heritier et al., 1994). During the trials, the subject would breathe through a mouthpiece (FreeFlow, Alpha MedTech Limited) connected to flow/pressure transducers sampling data at 1000 Hz (Bidirectional pneumotach, mod. 3830, Hans Rudolph), (Pressure box HF, KleisTek).

### 2.4 Magnetic stimulation

PI_max_ was determined using CMS (Magstim 200 Mono pulse, The MAGSTIM Company Limited). Stimulation intensity was set to 100% output, and single‐burst impulses were delivered by a circular 90 mm coil centered over the C7 (confirmed by palpation) (American Thoracic Society/European Respiratory Society, 2002).

### 2.5 Metabolic measurements

Fractions of O_2_ (FO_2_) and CO_2_ (FCO_2_) were measured side stream and sampled at 50 Hz (Beacon, Mermaid Care). The breathing circuit was checked for leaks by occluding the system and adding both negative and positive pressure before and after each trial.

VO_2_ and VCO_2_ were calculated breath by breath as the time integral of flow and the fractions of in‐ and expired gases. The flow was sampled at 1000 Hz, as described earlier, and therefore was down‐sampled to 50 Hz. Expiratory fractions of gas and volumes were converted to atmospheric temperature and pressure dry.

### 2.6 Ratings of perceived exertion

Subjects reported the modified Borg dyspnea scale (0–10) every 5 min by pointing on a paper clearly showing the scale.

### 2.7 Data analysis

The respiratory muscle endurance time (*T*_lim_) was established post hoc by examining the last 30% of the individual trial. *T*_lim_ was defined as the point in time where the following criteria were evident: Subjects reported RPE > 7 and at least three consecutive breaths where (*P*_es_ − *P*_trace_)/*P*_trace_ > ±15% of target pressure for >50% of *T*_i_ indicating inability to follow the inspiratory trace. *P*_trace_ was the exact trace the subject had to visually follow with the *P*_es_ (see Figure 2).

TTI_es_ was calculated breath by breath as ${\mathrm{TTI}}_{\mathrm{es}}=\frac{P_{\mathrm{es}}\left(\text{insp}\right)}{{\mathrm{PI}}_{\max}}\times \frac{T_{i}}{T_{\mathrm{tot}}}$ where *P*_es_(insp) was the mean of the *P*_es_ in the inspiratory phase, PI_max_ was measured before trial initiation, *T*_i_ was inspiratory time in seconds, and *T*_tot_ was total breath time in seconds.

TTI_es_, VO_2_, VCO_2_, end‐tidal fraction of CO_2_ (F_et_CO_2_), respiratory exchange ratio (RER), Borg, and *V*_t_ were calculated as mean of 2 min measurements at 0%, 50%, and 100% of *T*_lim_.

PI_max_ was noted as the highest peak of the six attempts and compared pre‐ and posttrial.

All data analysis was performed with MatLab (R2022a, MathWorks).

### 2.8 Statistics

A power calculation was performed based on previously observed *T*_lim_ at an approximate TTI_es_ of 0.25 where *T*_lim_ of 25–35 min was expected (Bellemare & Grassino, 1982). A conservative estimate of *T*_lim_ SD was set to be 5 min (assuming normal distribution). Assuming the same SD in both pressure waveform experiments, a sample size of 10 subjects was required for a paired *t*‐test to detect a 5 min difference in *T*_lim_ between two breathing modalities with a power of 0.8 at a level of significance of *p* < 0.05. Twelve subjects were decided to accommodate for measurement error and possible dropout. The power analysis was performed with G'power (Faul et al., 2007).

To test for difference between square wave breathing and triangle wave breathing in *T*_lim_, *P*_es_, *P*_di_, metabolism, perceived effort, and PI_max_ both between modality and within trial, the following statistical procedures were conducted.

Data were initially checked for normality with a Shapiro–Wilk test. For *T*_lim_, a paired *t*‐test was performed, and for PI_max_, VO_2_, VCO_2_, RER, *V*_t_, F_et_CO_2_, Borg, TTI, *P*_es,_ and *P*_di,_ ANOVA two‐way repeated measures was applied with time as repeated factor, and square/triangle as between groups factor. Bonferroni corrected post hoc tests were performed for all significant repeated measures tests. Statistical significance was considered at two‐tailed *p* < 0.05. All statistical procedures were carried out using SPSS (version 28, IBM).

## 3 RESULTS

Four subjects were excluded. Of these, three subjects withdrew from the experiment prematurely and one subject experienced vasovagal syncope as a result of the catheter insertion and was therefore excluded.

The flow signal was corrupted, potentially by water droplets in a sampling tube, in one subject. Therefore *N* = 7 for VO_2_, VCO_2_ F_et_CO_2_ and RER. *N* = 8 for all other parameters (*W* = 76 ± 10 kg, *H* = 181 ± 7.9 cm, age = 33.5 ± 4.8 years, sex [F/M] = 1/7).

**FIGURE 4:**
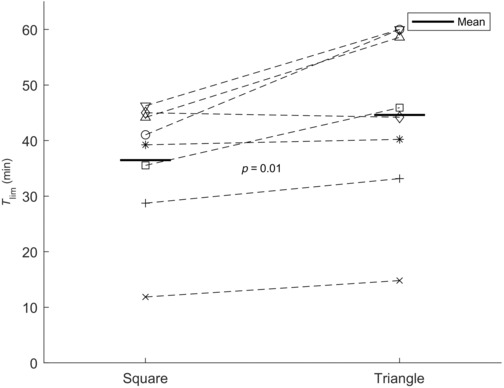
Mean *T*_lim_ for square wave (left side) and triangle (right side) for the eight subjects. The mean *T*_lim_ is signified with a horizontal black line. Each subject is represented by a unique symbol and the two different trial measurements are connected with a dashed line. All but one subject had higher *T*_lim_ during triangle wave breathing compared with square wave breathing.

Average respiratory muscle endurance was greater for triangle wave breathing compared with square wave breathing with a mean (SD) of 36.4 ± 11.2 min for square wave and 44.6 ± 15.6 min for triangle wave breathing, respectively. The mean difference in *T*_lim_ from square to triangle wave breathing was 8 ± 7.2 min (*p* = 0.015), see Figure 4.

**FIGURE 5:**
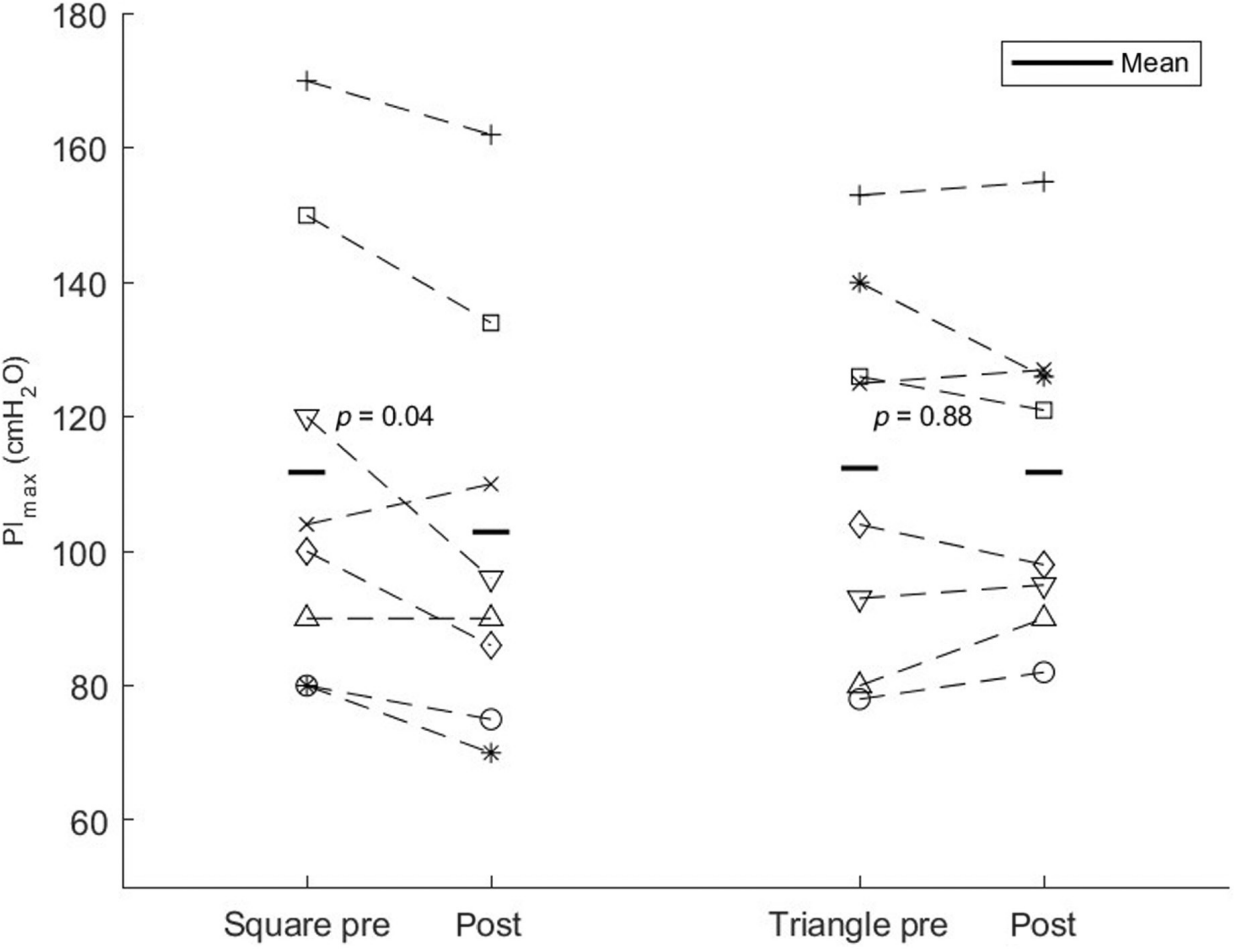
Horizontal black line indicates group mean of PI_max_ measured as the highest individual peak pre and post both square wave and triangle wave for the eight subjects. Each subject is represented by the same unique symbol as in Figure 4, and the pre‐ and postmeasurements of PI_max_ are connected with a dashed line. There was a significant reduction in PI_max_ from pre to post within the square wave breathing trial (*p* = 0.04).

A reduction was observed in PI_max_ for square wave breathing pattern (*p* = 0.04) but not for triangle wave breathing (*p* = 0.88), see Figure 5. The PI_max_ mean (SD) difference from pre to post for square wave breathing was −8.8 ± 9.4 cmH_2_O and −0.6 ± 7.3 cmH_2_O for triangle wave breathing.

*P*_es_ and *P*_di_ measured at 0%, 50%, and 100% showed no difference in time (*p* = 0.33 and *p* = 0.69). Mean difference from *P*_es_ to *P*_di_ was 10, 9, and 8 cmH_2_O for square wave breathing while 7, 8, and 7 cmH_2_O for triangle wave breathing. The difference between *P*_es_ and *P*_di_ was relatively constant during trials, varying less than was 2–6 cmH_2_O for square wave breathing and 3–4 cmH_2_O for triangle wave breathing trials.

Borg ratings of perceived exertion increased significantly from 0% to 50%, 50% to 100%, and from 0% to 100% within both square wave breathing (*p* = 0.006, *p* = 0.002, and *p* = 0.001) and triangle wave breathing (*p* = 0.01, *p* = 0.001, and *p* < 0.001) (see Table 1). No change was detected in Borg rating between square and triangle wave breathing at 0% (*p* = 0.27), 50% (*p* = 0.35), or 100% (*p* = 0.1).

**TABLE 1:** Mean ± SD for VO_2_, VCO_2_, F_et_CO_2_, RER, *V*_t_, TTI, and Borg rating from 0%, 50%, and 100% of *T*_lim_. For VO_2_, VCO_2_, FetCO_2,_ and RER, *n* = 7; for *V*_t_, TTI, and Borg rating, *n* = 8.

	0% square	0% triangle	50% square	50% triangle	100% square	100% triangle
Borg rating (0–10)	4.5 ± 1.6	4.0 ± 1.6	6.6 ± 1.0^b^	6.2 ± 1.0^d^	8.4 ± 0.5^c^	7.9 ± 0.8^e^
VO_2_ mL/min	493 ± 254^a^	689 ± 233	731 ± 259^b^	872 ± 245^d^	735 ± 283^a^ ^,^ ^c^	913 ± 255^e^
VCO_2_ mL/min	417 ± 143	476 ± 78	558 ± 162	627 ± 88^d^	613 ± 161	668 ± 156^e^
F_et_CO_2_%	4.7 ± 0.7	4.7 ± 0.6	4.9 ± 0.7	4.8 ± 0.6	4.9 ± 0.7	4.9 ± 0.6
RER (VCO_2_/VO_2_)	0.91 ± 0.3	0.75 ± 0.2	0.86 ± 0.3	0.75 ± 0.2	0.88 ± 0.2	0.76 ± 0.2
*V*_t_ mL	1151 ± 133	1134 ± 141	1201 ± 127	1263 ± 219	1312 ± 240	1292 ± 267
TTI	0.21 ± 0.02	0.21 ± 0.02	0.19 ± 0.02	0.20 ± 0.04	0.20 ± 0.01	0.19 ± 0.02

^a^ *p* < 0.05 between modality.

^b^ *p* < 0.05 within modality for square wave between 0% and 50%.

^c^ *p* < 0.05 between 0% and 100%.

^d^ *p* < 0.05 within modality for triangle wave between 0% and 50%.

^e^ *p* < 0.05 between 0% and 100%.

VO_2_ increased significantly from 0% to 50% and 0% to 100% but not from 50% to 100% within both square wave breathing (*p* = 0.02, *p* = 0.04, and *p* = 1.0) and triangle wave breathing (*p* = 0.01, *p* = 0.007, and *p* = 0.94) (see Table 1). Higher VO_2_ was observed during triangle wave breathing compared with square wave breathing at 0% and 100% (*p* = 0.039 and *p* = 0.048), but not at 50% (*p* = 0.11).

VCO_2_ increased significantly from 0% to 50% and 0% to 100% but not from 50% to 100% within triangle wave breathing (*p* = 0.002, *p* = 0.039, and *p* = 1.0) (see Table 1). An increasing trend for VCO_2_ but no difference was observed for square wave breathing from 0% to 50%, 0% to 100%, and 50% to 100% (*p* = 0.09, *p* = 0.06, and *p* = 1.0) (see Table 1). No change in VCO_2_ was detected between square and triangle wave breathing at 0% (*p* = 0.14), 50% (*p* = 0.31), or 100% (*p* = 0.48).

There was an increasing trend for *V*_t_ with a significant within‐trial change (*p* = 0.02), but post hoc tests revealed no significant differences in *V*_t_ for either square wave breathing or triangle wave breathing at 0% to 50% (*p* = 0.48, *p* = 0.11), 0% to 100% (*p* = 0.48, *p* = 0.19), or 50% to 100% (*p* = 0.24, *p* = 1.0) (see Table 1).

There was no effect of time in F_et_CO_2_, RER, or TTI (*p* = 0.4, *p* = 0.88, and *p* = 0.53, respectively) (see Table 1).

## 4 DISCUSSION

Previous work investigating RMF during inspiratory loaded breathing has to the best of the authors' knowledge exclusively applied square wave breathing. This study investigated respiratory muscle endurance during inspiratory loaded breathing comparing square wave breathing to triangle wave breathing. The major finding of this study was that respiratory muscle endurance (*T*_lim_) was significantly lower during square wave breathing compared with triangle wave breathing under matching breathing frequency, duty cycle, and average load. RMF did occur after square wave breathing but was not present after triangle wave breathing shown by the significant reduction in maximal pressure generation capacity after square wave breathing. There was an increasing metabolism throughout the trial with significantly higher VO_2_ during triangle wave breathing compared with square wave breathing in the beginning and end of the trial. The subjects reported increasing perceived effort of breathing as the trial progressed, but with no difference between the breathing patterns. Overall, these are the first results showing the effect of inspiratory loading with a non‐square breathing pattern and demonstrate that one cannot directly transfer findings from square wave breathing to the more natural triangle breathing pattern.

### 4.1 *T*_lim_ , PI_max,_ and perceived effort

In the current study, the mean TTI was 0.21 and following the isopleth model from Bellemare and Grassino (1982) would thus predict a *T*_lim_ of about 35 min. Furthermore, a mean loading of 50% PI_max_, as used in the current study, would predict a *T*_lim_ well within 30 min according to the data provided in the study by Roussos and Macklem (1977). The current *T*_lim_ was 36 and 44 min for square wave and triangle wave breathing with two of eight subjects stopped at 60 min during triangle wave breathing as RMF was deemed not reachable. As such, the findings replicated those of Bellemare and Grassino for square wave breathing. However, introducing a triangle wave breathing pattern led to significantly longer *T*_lim_ compared with square wave breathing in seven out of eight subjects with an average within‐subject increase in *T*_lim_ of 9.5 ± 6.7 min up to a maximum of 19 min. This new finding indicates a significant and important role of the inspiratory pressure pattern of breathing in determining diaphragm endurance.

Square wave breathing was followed by a significant reduction in PI_max_ confirming the presence of RMF during this breathing pattern (Workshop, 1990). This reduction is in line with previous studies using inspiratory loading of 50%–60% of PI_max_ and a duty cycle of 0.5 (Bezzi et al., 2003; Delpech et al., 2003). They showed pre‐ to postreductions in esophageal PI_max_ with CMS of 14% to 18% (Bezzi et al., 2003; Delpech et al., 2003). Some studies have showed RMF and PI_max_ reductions of up to 60% following RMF at inspiratory loading of 50% of PI_max_ (Janssens et al., 2013). This discrepancy may be due to differences in the loading protocol such as higher duty cycle and different PI_max_ measurement methods used for targeting load as well as evaluating RMF. In contrast, PI_max_ was not reduced significantly following triangle breathing pattern corroborating that diaphragm endurance was improved in this breathing pattern as compared to square wave breathing. Previously, respiratory pattern has only been considered via duty cycle and average load. Both of these were controlled for in the present study by controlling for TTI. As shown in Table 1 and as described in the results section, *T*_i_/*T*_tot,_ and *P*_es_ were indeed maintained constant during breathing trials. As expected, the perceived effort increased in both breathing patterns as the trial progressed. At *T*_lim_, the Borg ratings ranged from “severe” to “extremely severe” for all subjects regardless of the breathing pattern. Slightly higher ratings were observed throughout the square wave trial as compared to the triangle wave trial which indicates that the subjects had the perception of working harder during the square wave breathing trial. This is underlining that RMF is more prone to occur in one type of pressure waveform but to a lesser degree in another. Therefore, the pressure waveform is highly relevant to consider, when judging if RMF will occur or not.

### 4.2 Motor units and time under tension

**FIGURE 6:**
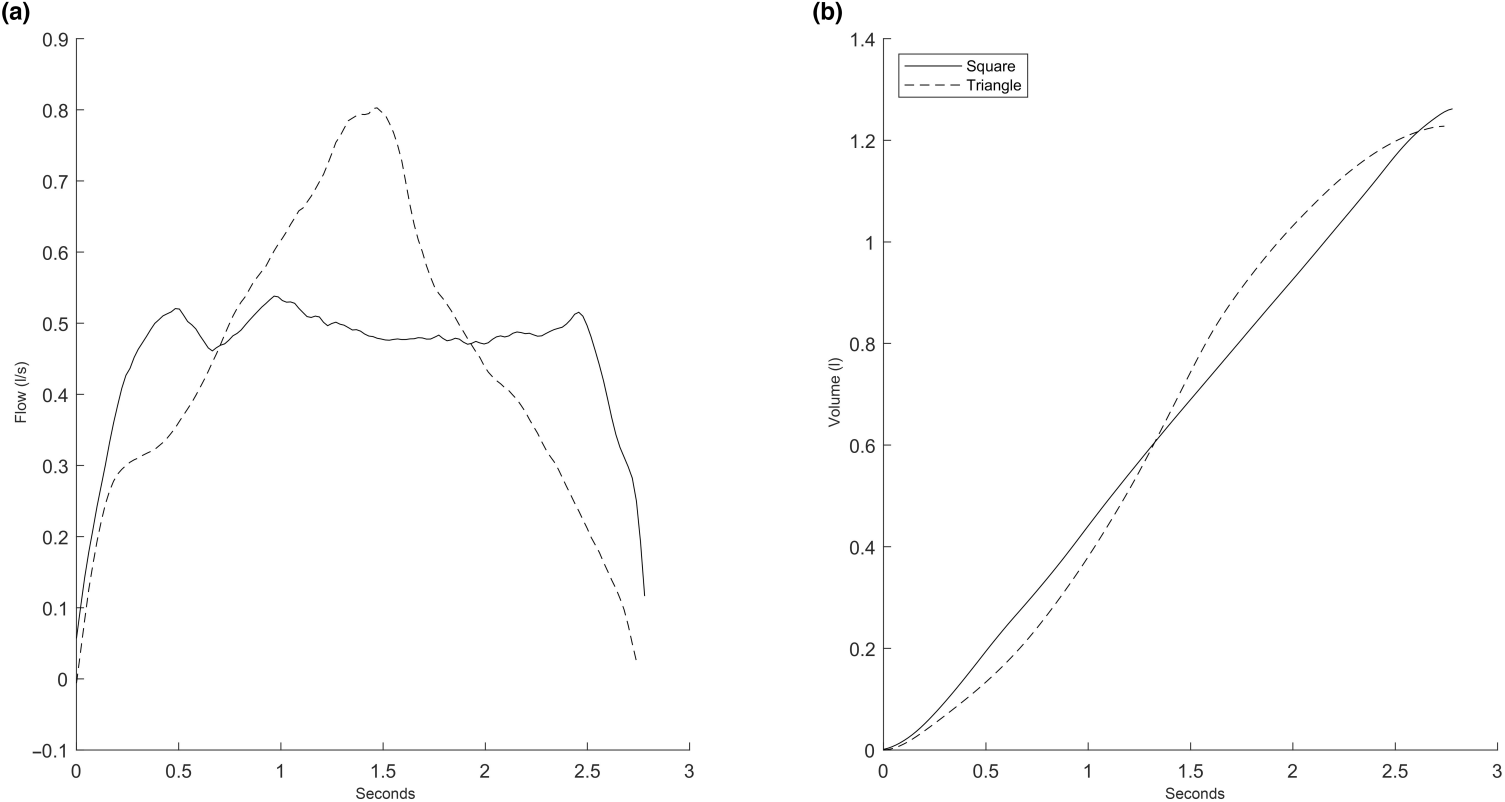
The flow (a) and cumulative volume (b) of one breath from a typical subject during square and triangle wave breathing. The solid line indicates square wave breathing whereas the dashed line indicates triangle wave breathing.

It is likely that the differences in endurance and fatigue in different breathing patterns could be due to triangular wave breathing. Triangle wave breathing could potentially be drawing on a wider range of diaphragm motor units as this type of contraction likely follows Hennemann's size principle with smaller motor units recruited first, then as the force required to follow the guide trace is increasing, larger and larger motor units are recruited (Henneman & Olson, 1965). This theory is consistent with the trend of cumulative volume observed in the inspiratory phase from square to triangle wave breathing, as exemplified in Figure 6. During square wave breathing, volume accumulates linearly whereas the accumulation was “s‐shaped” during triangle wave breathing. This could indicate that under triangle wave breathing large motor units have less time under tension but are responsible for a significant part of the volume accumulation. Triangle wave breathing might therefore be a more fatigue‐resistant breathing pattern as the main flow‐driving motor units are recruited for shorter intervals allowing longer restitution, that is, a lower duty cycle, for these specific motor units.

### 4.3 Metabolism and TTI

Assuming that the mechanical work between square and triangle breathing was the same, as the mean pressure and *V*_t_ were matched, the finding of higher VO_2_ during triangle wave breathing was surprising, as the higher VO_2_ could indicate worse efficiency during this breathing pattern in contrast to the findings in *T*_lim_, PI_max,_ and perceived exertion. Previously, Collett et al. (1985) investigated the efficiency of breathing by calculating work of breathing and measuring metabolic cost of breathing, at increasing breathing loads, finding that efficiency remained constant over a TTI range of 0 to >0.28. Our data suggest that there might be an efficiency difference depending on the pressure waveform despite matching of delivered mechanical work between the square wave and triangle wave breathing modalities.

Field et al. (1984) found a good correlation (*r* = 0.74) between TTI and VO_2_ in four healthy subjects during varying load breathing. Furthermore, McCool et al. (1989) investigated VO_2_ at various inspiratory resistances in four healthy subjects and showed that VO_2_ was positively associated with increased respiratory work and therefore concluded that VO_2_ possibly was the governing factor determining *T*_lim_ at different breathing tasks. The increased respiratory muscle endurance but with coinciding higher VO_2_ during triangle wave breathing might indicate that VO_2_ per se is not the determinant of *T*_lim_, as previously discussed by these authors (McCool et al., 1989; Roussos & Macklem, 1977). It has been proposed that *T*_lim_ was affected by anaerobic metabolism at high breathing loads caused by a restriction of blood flow to the respiratory muscles (Bellemare & Grassino, 1982). Results have not previously been able to confirm this (Collett et al., 1985). Indeed, the trend of increased RER during square wave breathing from the present study does indicate that anaerobic metabolism might play a role in a shortened respiratory muscle endurance during square wave breathing. Furthermore, the higher VO_2_ during triangle wave breathing despite increased *T*_lim_ might be a result of the short period of high respiratory muscle tension, leaving a longer period of lower respiratory muscle tension resulting in better conditions for perfusion of the capillaries. In other words, the triangle wave breathing potentially enables a better supply of oxygen to meet demand.

### 4.4 Limitations

The study had several limitations. One specific *P*_di_/PI_max_ and fixed *T*_i_/*T*_tot_ was applied, resulting in fixed TTI. This was to analyze the effects of the two inspiratory pressure waveforms at a corresponding and equal average TTI to control for duty cycle and relative mean load. An important assumption is here that it is appropriate to control for average *P*_I_/PI_max_ in each breathing modality.

The participants followed a guide trace from *P*_es_. Guiding the subjects by *P*_di_ would be preferable in a future study, however, the two signals were well aligned with the difference during a trial staying within 2–6 cmH_2_O.

Observed values of *T*_lim_ showed a relatively high intersubject variation (Figure 4). However, an average *T*_lim_ of 36 min was within the expected range according to the isopleths of *T*_lim_ vs inspiratory load reported previously by Bellemare and Grassino (1982). The range of *T*_lim_ for square wave breathing in the present study is larger than reported previously in a similar inspiratory loading protocol where a *T*_lim_ range of 12–34 min was reported during loading at 60% PI_max_ (Bezzi et al., 2003). It is evident from Figure 4 that the subject depicted by “*x*” in Figures 4 and 5 contributed considerably to the range of observed *T*_lim_. Excluding this subject would result in a range of *T*_lim_ within 20 min, in line with the range reported by Bezzi et al. However, there were no apparent reasons for excluding this subject, whose PI_max_ was around the overall average, and whose measurements of *T*_lim_ were consistently low compared with other subjects in both square and triangle breathing trials. Reproducibility of measurements might have influenced variability of *T*_lim_. It is not possible, however, to discern to what degree this might have had a role in the present study. Subjects underwent one breathing trial in each loading mode, leaving no room for analyzing the coefficient of variation within a patient under the same breathing modality. The protocol was kept at few breathing trials to minimize subject discomfort and focus on the role of inspiratory loading pattern. It would, however, be relevant in future studies to investigate the within‐subject variability in respiratory muscle endurance during inspiratory loading as well the contribution of breathing pattern to this variability.

The importance of the inspiratory pressure waveform with regard to muscle endurance may depend on lung mechanics and capacities. Subjects were between 25 and 37 years and with a BMI between 21 and 25. Older subjects may have increased lung compliance and consequently slightly increased FRC (Kim et al., 2017), where, for example, obese subjects have opposite characteristics (Mafort et al., 2016). It would be interesting to see whether the results observed in the present study would translate to a broader test population.

The authors would argue that a triangle inspiratory pressure waveform more closely reflects a natural breathing pattern as compared to a square wave pattern. However, we did not investigate fully natural breathing with associated breath‐to‐breath variations in depth, duty cycle, etc. It remains to be seen whether subjects during a self‐selected breathing pattern would elicit levels of endurance reflecting those observed in the present study and to what degree such results would reflect those for triangle wave breathing pattern.

PI_max_ was measured with Mueller maneuvers as this is an accepted test to perform maximal inspiratory contractions of the diaphragm (American Thoracic Society/European Respiratory Society, 2002). Using Mueller expulsive maneuvers have been shown to produce higher pressures, but we chose to refrain from this as the maneuver requires much training and has been described difficult for subjects to perform. To ensure that the subjects were reaching PI_max_, maximal cervical magnetic twitch stimulation, on top of a maximal inspiratory effort was conducted. Furthermore, the same method was used, regardless of breathing modality, so the differences observed have occurred from trials performed under similar conditions.

## 5 CONCLUSION

The current study showed that *T*_lim_ was significantly lower in square wave breathing than in triangle wave breathing at equal inspiratory load, which shows that the pressure waveform has a high impact on the function and endurance of the respiratory muscles. Furthermore, the study shows that prediction of *T*_lim_ from previous indexes requires further interpretation. Surprisingly, VO_2_ was significantly higher during triangle wave breathing, indicating a worse mechanical efficiency, but both PI_max_, RER, and Borg ratings showed trends of this modality being less stressful for the subjects.

## FUNDING INFORMATION

No funding information provided.

## CONFLICT OF INTEREST STATEMENT

Søren Kjærgaard, Stephen E. Rees, and Dan S. Karbing are minor shareholders of Mermaid Care A/S, but as agreed before the study and stated in the ethical approval, results from the current study would be sought published no matter how the result came out. The study was entirely funded by Aalborg University.

## ETHICS STATEMENT

The study including all experimental procedures was approved by the local ethical committee of the North Denmark Region (N‐20210005) and adhered to the Declaration of Helsinki II. The study was registered on ClinicalTrials.gov (NCT05393115).
